# Boldness Predicts Social Status in Zebrafish (*Danio rerio*)

**DOI:** 10.1371/journal.pone.0023565

**Published:** 2011-08-17

**Authors:** S. Josefin Dahlbom, David Lagman, Katrin Lundstedt-Enkel, L. Fredrik Sundström, Svante Winberg

**Affiliations:** 1 Department of Neuroscience, Uppsala University, Uppsala, Sweden; 2 Department of Organismal Biology, Uppsala University, Uppsala, Sweden; 3 Department of Animal Ecology, Uppsala University, Uppsala, Sweden; Cajal Institute, Consejo Superior de Investigaciones Científicas, Spain

## Abstract

This study explored if boldness could be used to predict social status. First, boldness was assessed by monitoring individual zebrafish behaviour in (1) an unfamiliar barren environment with no shelter (open field), (2) the same environment when a roof was introduced as a shelter, and (3) when the roof was removed and an unfamiliar object (Lego® brick) was introduced. Next, after a resting period of minimum one week, social status of the fish was determined in a dyadic contest and dominant/subordinate individuals were determined as the winner/loser of two consecutive contests. Multivariate data analyses showed that males were bolder than females and that the behaviours expressed by the fish during the boldness tests could be used to predict which fish would later become dominant and subordinate in the ensuing dyadic contest. We conclude that bold behaviour is positively correlated to dominance in zebrafish and that boldness is not solely a consequence of social dominance.

## Introduction

All animals are faced with challenges that can be approached in mainly two ways, each defining a ‘coping style’. *Proactive* animals react actively to threatening situations by fleeing or attacking and are considered to be bold. *Reactive* animals are more careful and prefer to wait passively for a threat to pass, and are therefore considered to be shy [1], [2]. In addition, reactive animals respond to stress with higher activation of the hypothalamic-pituitary-adrenal (HPA)/interrenal (HPI, the interrenal being the teleostean homologue of the mammalian adrenal) axis, leading to higher post stress levels of glucocorticoids in reactive than proactive animals [1], [3], [4]. These coping styles have been found repeatedly in rodents [1], [3], [4] and fish, such as three-spined stickleback (*Gasterosteus aculeatus*) [5], [6], [7], brown trout (*Salmo trutta*) [8], Senegalese sole (*Solea senegalensis*) [9] and rainbow trout (*Oncorhynchus mykiss*) [10]. The proactive strategy can increase survival by gaining access to better food resources and proactive animals have been shown to sire more offspring under certain circumstances [1], [8], [11], [12], [13]. In contrast, reactive animals act passively towards threats, thus surviving by staying away from danger.

These coping styles are closely linked to social status. Proactive animals are more aggressive and often become dominant over reactive ones [1], [8], [11], [12], [13]. Being subordinate is highly stressful and leads to elevated plasma cortisol levels and, in severe cases, anorexia or even death in laboratory settings where there is limited possibility for escape [14], [15]. Bernier and colleagues [16] concluded that the physiological stress response activated during the formation and maintenance of a dominant- subordinate relationship in rainbow trout was much more severe than when exposed to hypoxia, ammonia, isolation or human handling. Thus, the stress perceived and the physiological responses are both context and stressor specific. Moreover, the response to stress is also highly individual and does not necessarily have to be consistent between different kinds of stressors. For example, two lines of rainbow trout, selectively bred for high or low HPI-axis response to confinement stress (reactive and proactive coping styles, respectively), have been established and their behaviour is well studied [17]. Pottinger and Carrick [13] showed that the second generation of these trout differed in their probability to become dominant. They found that the low responsive (LR) line became dominant significantly more often than high responsive (HR) line in dyadic fights [13]. In contrast, Schjolden *et al.* found the same generation of HR fish to be more aggressive than LR fish against an intruder from the HR line [18]. In addition, Coleman and Wilson [19] have shown that pumpkinseed sunfish (*Lepomis gibbosus*) which were bold in approaching a novel metre-stick were not more likely to feed from a novel source than fish that had fled from the stick.

Anxious, also referred to as shy, behaviour in zebrafish (*Danio rerio*) has been studied in different types of tests, which are usually evaluated with various anxiolytic and anxiogenic substances [20], [21], [ review by 22]. Some of the most commonly studied behaviours that are associated with a shy behavioural profile in zebrafish are freezing (movement of only the opercula and pectoral fins while the rest of the body is still), erratic movement (fast movement with sharp turning angles) and thigmotaxis (preference to stay close to walls of the arena, rather than explore the middle) [23]. In contrast, general activity, measured as distance moved, as well as inspection of predators and novel objects are more pronounced in bolder animals [23].

So far, studies on coping styles at individual level are limited in zebrafish. However, Moretz and colleagues showed a correlation between activity and the tendency to approach a predator dummy [24]. In addition, it has been shown that zebrafish treated with low doses of alcohol display more aggressive behaviours towards a mirror [25] and spend less time at the bottom of a novel tank, both being indicative of increased boldness [26]. Though these traits were not tested together, it provides an indication that aggression and bold behaviour are also likely to be related in zebrafish.

The zebrafish is a shoaling species but has been shown to act aggressively and develop clear dominance- subordinate relationships when kept in pairs [27], [28], [29]. The primary aim of this study was to explore whether personality traits in preceding behaviour tests could predict the outcome of social fights. We found that animals displaying bolder behaviour in the tests were more likely to become dominant than animals displaying a shyer behavioural profile. The secondary aim was to explore whether males and females differed in their behavioural profiles and indeed, males were found to be bolder than females.

## Materials and Methods

### 1. Animals and tagging

Adult zebrafish wild-caught from North Bengal, India (Dogman, Sweden) were held in the lab for 15 months before experiments were conducted. Fish were housed in an Aquaneering Zebrafish system with light∶ dark cycles of 14∶10 hrs at 27°C in Uppsala municipality tap water (pH 7.2–7.6) of which 15% was exchanged daily. Fish were sorted by sex, anaesthetized in benzocain (ethyl p-aminobenzoate, 0.34 mg/ml), weighed, measured for standard length (tip of the snout to base of the caudal fin) and tagged. Tagging was done by pulling a 0.4 mm needle with a 0.15 mm diameter nylon monofilament through the dorsal musculature, removing the needle but leaving the filament, melting the ends of the filament and painting them with nail polish in different colour combinations. Tagging did not affect swimming patterns in home tanks and all fish ate within one hour after the procedure. Fish were housed individually in 2.8 l tanks for at least one week before any experiments started. Main feed was Sera San tropical flake food, which was provided once daily (Gibbon, Sweden).

The experimental protocols and use of animals in this research was approved by Uppsala Ethical Committee (Permit number: C 33/10).

### 2. Testing for boldness

Prior to the boldness test, fish were housed in isolation for at least one week before being transferred from their home aquarium to the experimental arena where they were released into a black circular enclosure (diameter 7 cm) placed in a corner of the arena. After 30 s of acclimation, the enclosure was carefully removed and tracking of the fish using Ethovision 3.0 (Noldus, The Netherlands) started.

**Figure 1:**
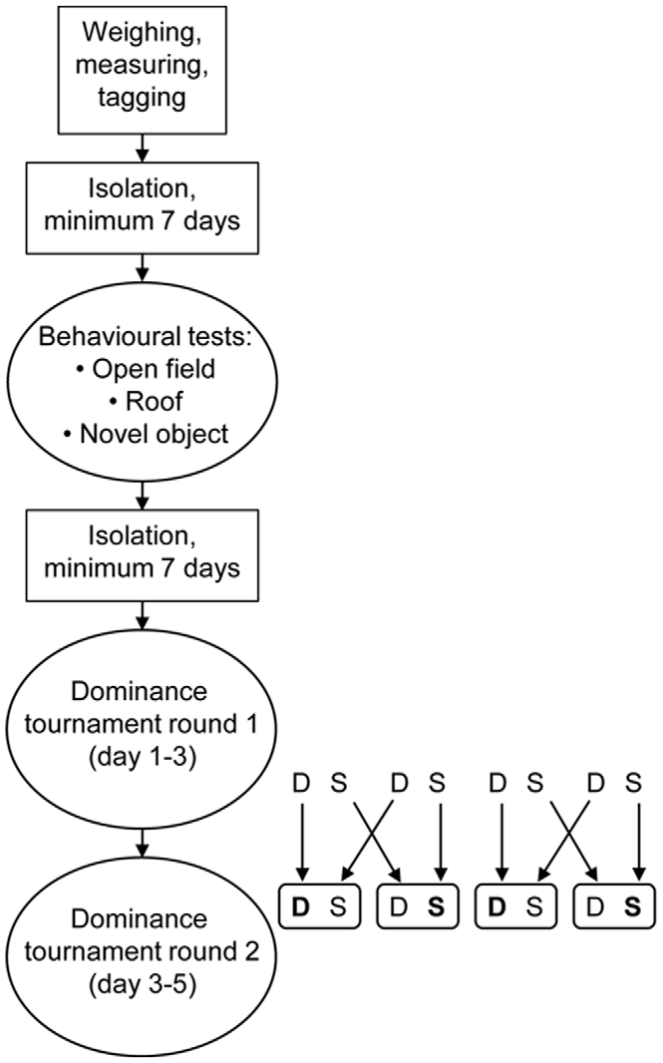
Experimental setup. Zebrafish were screened for boldness in three behavioural tests; open field, roof (addition of a shelter to the open field) and novel object (removing the roof and adding a white Lego® brick at the centre). After a minimum of one week, a tournament was set up in two rounds to generate animals that twice obtained the same social status. After two rounds, there were eight extremes from each social status and these were used for subsequent analyses.

Three behavioural tests were performed: open field, roof (shelter seeking) and novel object. These tests have all been used previously when testing for boldness in fish [23], [30]. High activity (distance moved), time spent out of a shelter and low thigmotaxis (staying close to the walls of the arena) are common measures indicating increased boldness in these tests [23], [30]. The three tests were performed consecutively in the same arena, each lasting for a total of 15 min, divided into three 5 min periods (period 1–3) (Figure 1). The first test, ‘open field’, consisted of releasing the fish in a novel, empty arena (29×19 cm filled to a depth of 2 cm (1.2 l) with system water at 25°C) that had a white semi-opaque bottom lit from underneath with two fluorescent lights (13 W). To minimize disturbance from the surroundings, a 20 cm high, dark **grey** wall was placed 5 cm from the long sides and 20 cm from the short sides of the arena. After 15 min, an opaque, **light blue**, piece of PVC (5×5 cm) was placed as a shelter on the water surface in a corner of the arena before tracking started again **(test 2, ‘roof’)**. After a further 15 min, the shelter was carefully removed and a novel object consisting of a white Lego® brick (2×4 dots, 8 mm high) was introduced into the centre of the test arena before tracking started again (test 3, ‘novel object’). In all tests, the distance moved was scored and in the open field and novel object tests **(1 and 3)**, the amount of time spent in the centre zone (ellipse of 4×7 cm) of the arena was quantified. In the roof test the amount of time spent hiding under the roof was scored.

After each fish, the arena was rinsed with warm tap water, sprayed with 70% ethanol, wiped and rinsed with system water before the next fish was studied. After the boldness test, fish were isolated for at least one week before the tournament began.

### 3. Tournament

***The relationship between boldness and dominance was explored by use of a tournament from which animals could be categorised as highly dominant or subordinate while at the same time avoiding*** intermediates. If boldness and social status were linked, as we hypothesised, this procedure would ***highlight*** extremes in boldness and shyness.

The tournament tanks were identical to the ones used for housing. In the first round of the tournament, fish were paired with one of the same sex and similar weight (mean weight difference ± sd for round 1 was 8.6%±15.4% and for round 2; 21.7%±14.1%). Fish were caught from their home aquaria and placed individually in 250 ml glass beakers filled with 200 ml system water and then, both at the same time, carefully poured into the tournament tank, which was novel to them both. During three days of fighting, daily observations were made three times per day to keep track of social rank and potential changes thereof. Dominant was defined as being the fish that patrolled the tank and performed the most attacks and chases. To make the dominance relationship more visible a small amount of food was given daily, with the assumption that the dominant fish would eat first and defend the food [31]. In the second round, fish were placed with a new competitor of the same social rank as determined during the first round, i.e. dominants met other dominants and subordinates met subordinates. As in the first round, weight-matched fish of same sex were moved individually into beakers and poured, both at the same time, into a tank novel to them both. As dominant fish did not defend the food in the first round, fish were not fed during the second round in order to increase aggression and thus make the hierarchy clearer. See figure 1 for tournament setup.

### 4.1. Statistical analysis

In order to reduce variance, behavioural data was normalised before analysis. Time spent in the zone in each period was normalised as follows: (time in zone (seconds)×100/total test time (i.e. 2700 s)). Similarly, the distance moved in each test and period was normalised as: (distance moved in one period×100/total distance moved in all three tests).

The relationship between social rank and boldness was analysed using the multivariate method partial least square (projection to latent structures) discriminant analysis (PLS-DA) [33]. PLS-DA is a regression extension of principal component analysis (PCA) and calculates the relationship between a Y-matrix and an X-matrix. The Y-matrix that in PLS consist of the response variables consists in PLS-DA of categorical so-called binary dummy variables (dominant/subordinate as one/zero in this study) while the X-matrix contains the predictor variables (behaviours, in this study). The Y is then modelled versus the X-matrix in order to identify which variable/-s (if any) are important for the separation of the two categories [34]. The relationship between sex and boldness was analysed using the same method with male/female being set as the Y-matrix (one/zero respectively).

For multivariate data analyses the software Simca-P+ 12.0 (Umetrics AB, Umeå, Sweden) was used. To study only the most important variation when performing the PLS-DA, components were extracted as long as eigenvalues were greater than two, or, if there were significant components (determined using cross validation) [32] with eigenvalues lower than two.

Univariate and bivariate analyses were performed with the software PASW Statistics 18.

### 4.2. Sex differences

To examine differences in behaviour between the sexes, the variables *male* and *female* (as binary variables) were set as Y while the behavioural variables from the three 5 min periods (i.e. not including the sum of the periods) were set as X in the PLS-DA. Data from all animals were used (N = 32).

### 4.3. Social status

Only behavioural data from animals that became dominant or subordinate, respectively in both rounds of the tournament were used for analysis (N = 8 dominant, N = 7 subordinate as one pair had to be excluded, see results, section 3.1).

A PLS-DA was performed with *dominant* and *subordinate* as binary variables, Y. The model creates an equation that can be used to calculate each individual's Y-value (social status) based on its X-values (behaviour) as the model quantifies the relationships between behaviours in the three preceding tests and the fish result in the tournament later. As the variable *dominant* was set to 1 and *subordinate* was set to 0, predictions for a fish above 0.5 were interpreted as that fish being dominant and predictions below 0.5 were interpreted as that fish being subordinate. The calculated Y-values obtained were used in a t-test to investigate if the model could significantly separate dominants and subordinates. To establish which behaviours were associated with which social status, the loadings of each behaviour together with the 95% confidence interval for the respective variables, were investigated.

To investigate how stable the model was, we explored if shorter experimental time would be sufficient to calculate the actual social status, starting with the first period alone and then the first two periods combined, comparing with the results when using all three periods.

### 4.4. Relationship between behaviours

To give an overview of variables that may cluster, a principal component analysis (PCA) was performed with all individuals (N = 32). As this grouped the periods within the behaviours, Pearson correlation analysis was performed with the sum of the periods within the respective behaviours (i.e. 6 variables) to reduce the number of variables and thereby the risk of spurious results.

## Results

In all pairs, dominance hierarchies were established and there were no changes in social rank after the first day. In all cases, the dominant chased and bit the subordinate and in most cases, the subordinates lay at the bottom but in some cases, they lay on their side at the bottom where the dark back of the tank could possibly shield them. However, all subordinates still ate and the dominant fish did not defend the feed. In one case both fish swam freely throughout the entire tank but the dominant was still chasing the subordinate with a low frequency.

During the tournament, one female died in the second round where the outcome would generate an individual that would have become subordinate twice. At close examination, it appeared that the opponent had bit the tag, resulting in a large wound that most likely led to the mortal outcome. This pair was excluded from further analysis regarding social status but not regarding sex when only the previously determined boldness behavioural test results were used.

Erratic movement and freezing were not analysed with the software but by manual observation of the track data. No erratic movement appeared to occur, but there were several instances with very little movement for several minutes, which is likely to have been freezing.

The colour used on the tag had no effect on the outcome of the tournament, but fish with blue tags showed a tendency to be more likely to become dominant than subordinate (81% dominant) (Chi^2^, p = 0.051). There was no difference in weight between fish ending up as dominant or subordinate, comparing each individual pair in both rounds (N = 32, paired t-test, p = 0.196, mean weight (g) ± sd: dominant twice (N = 8) was 0.45±0.14, subordinate twice (N = 7) was 0.36±0.10) nor between males and females (t-test, p = 0.605, mean weight (g) ± sd: male (N = 16) was 0.40±0.10, female (N = 16) was 0.42±0.18).

### 1. Effects of sex

**Figure 2:**
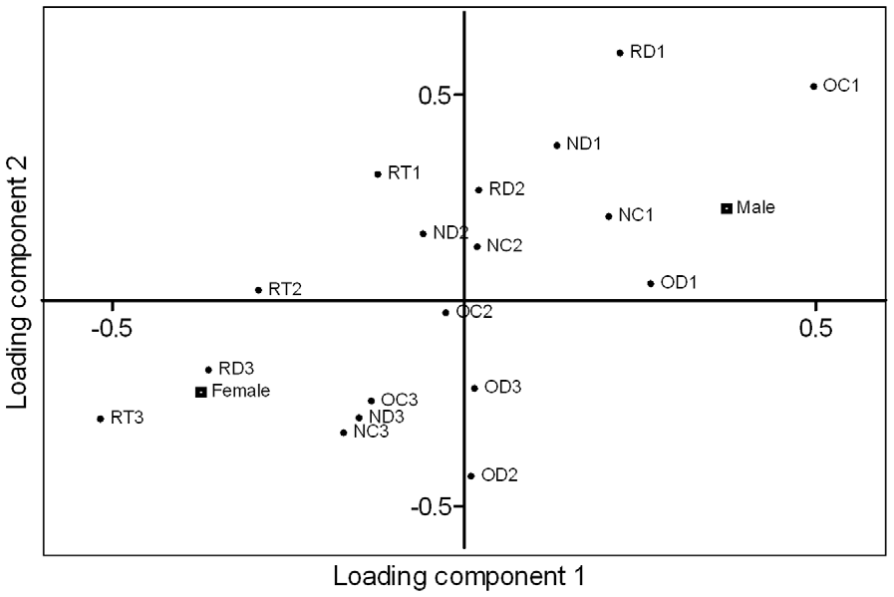
Behaviours associated with males and females. Scatter plot of variable loadings from PLS-DA (R^2^X = 0.353, R^2^Y = 0.417, Q^2^ = −0.0277, 2 components). *Male* and *female* as Y (binary variables) and the behaviours as X. Abbreviations: OD; distance moved in open field test, OC; time spent in the centre zone in the open field test, RD; distance moved in the roof test, RT; time spent underneath the roof in the roof test, ND; distance moved in the novel object test, NC; time spent in the centre zone in the novel object test. 1–3 denotes the three consecutive 5-minutes periods 1–3.

A PLS-DA with *male* and *female* set as binary variables (Y) extracted two components with eigenvalues larger than two and showed that males and females behaved differently (R^2^X = 0.353, R^2^Y = 0.417, Q^2^ = −0.0277). A scatter plot of the variable loadings showed that behaviours associated with females were expressed during the third period (Figure 2). Behaviours associated with males were instead expressed during the first period, meaning that the sexes differed in their timing of the behaviours. The variable loadings from the first two components showed that females spent more time under the roof particularly in the third period while males spent more time in the centre in the open field in the first period. Subsequent t-tests between females and males confirmed the difference in time spent under the roof in the third period (p = 0.040) and showed a tendency to differ in time spent in the centre of the open field (p = 0.051).

### 2. Prediction of social status

**Figure 3:**
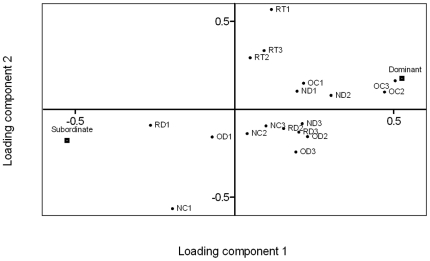
Behaviours associated with dominant and subordinate animals before social interaction. Scatter plot of the first two components of a PLS-DA (R^2^X = 0.563, R^2^Y = 0.861, Q^2^ = 0.220, 3 components) with dominant and subordinate individuals as Y (binary variables) and behaviours as X. See figure 2 legend for abbreviations.

A PLS-DA with *dominant* and *subordinate* as binary variables Y, and the behaviours as X, generated a model (R^2^X = 0.563, R^2^Y = 0.861, Q^2^ = 0.220, 3 components) that showed that animals that later became dominant had behaved differently in the boldness tests compared to fish that later became subordinate. The variable loadings scatter plot (Figure 3) showed that individuals that later became dominant spent more time in the centre zone in the second and third period in the open field test. This was confirmed with t-tests between dominant and subordinate fish (second period p = 0.008, third period p = 0.003). In addition, the variable loadings showed that dominant individuals moved more in the novel object test in the second period. Individuals that later became subordinate had a higher activity in the roof test during the first period and also spent more time in the centre in the novel object test during the first period (Figure 3).

**Figure 4:**
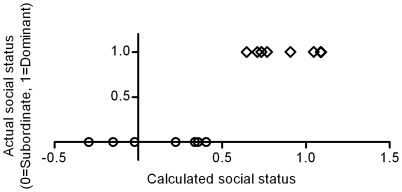
Observed vs. predicted social status. Scatter plot of actual social status (Y) and calculated social status based on behavioural profile (X) exhibited prior so social interaction. Modelled with PLS-DA (R2X = 0.563, R2Y = 0.861, Q^2^ = 0.220, 3 components).

A plot of the actual Y (dominant or subordinate) versus the calculated Y-value (what the model predicts) showed a total separation between the social statuses already in the first component (Figure 4). A t-test with the calculated Y-values confirmed this separation (p<0.001) meaning that all individual fish were correctly assigned their actual social status based on their behaviours in the boldness tests.

In the test of the stability of the model, to establish if a shorter time period was sufficient to discriminate dominants from subordinates, a PLS-DA using only values from the first five-minute period indicated that this could be sufficient to predict social status (R^2^X = 0.525, R^2^Y = 0.500, Q^2^ = −0.255, 2 components). A t-test differentiated between the calculated social ranks (p = 0.003) but one dominant and one subordinate were classified wrongly (calculated Y-value dominant = 0.47, calculated Y-value subordinate = 0.51). However, combining values from the first two periods separated dominant and subordinate individuals (PLS-DA, R^2^X = 0.544, R^2^Y = 0.895, Q^2^ = −0.0306, 3 components,) and a following t-test showed that the separation was complete (p<0.001), with no individual classified to the wrong status.

### 3. Relationships between behaviours

The PCA grouped the periods within each behaviour together (4 components). Relationships between the behaviours distance moved in open field, roof and novel object, time spent in the centre in open field and novel object as well as time spent underneath the roof are found in table 1. See Table S1 for performed behavioural acts.

**Table 1:** Pearson correlations between behaviour traits (above diagonal) and associated p-values (below diagonal).

Behaviour	OD	OC	RD	RT	ND	NC
OD	-	r = 0.310	r = −0.731	r = −0.455	r = −0.581	r = −0.095
OC	p = 0.090	-	r = −0.216	r = −0.202	r = −0.148	r = 0.367
RD	p<0.001*	p = 0.251	-	r = 0.407	r = 0.059	r = 0.162
RT	p = 0.010*	p = 0.284	p = 0.026*	-	r = −0.076	r = −0.125
ND	p<0.001*	p = 0.426	p = 0.752	p = 0.683	-	r = 0.033
NC	p = 0.616	p = 0.050	p = 0.401	p = 0.511	p = 0.863	-

Abbreviations: OD; distance moved in open field test, OC; time spent in the centre zone in the open field test, RD; distance moved in the roof test, RT; time spent underneath the roof in the roof test, ND; distance moved in the novel object test, NC; time spent in the centre zone in the novel object test.

*denotes significance at the p<0.05 level.

## Discussion

In this study, fish were screened for boldness, a personality trait related to coping style [33], prior to pair-wise social interaction. Dominant and subordinate animals showed very different personality traits in their behaviour, even before the social encounter where their social status was determined. Further, our multivariate model assigned fish the correct social rank from the behavioural profile expressed earlier. This implies that bold behaviour is not solely a consequence of social interactions, but rather that boldness and social dominance may have a common genetic basis. We found that dominant individuals' behaviour was associated with boldness, but only during the last two periods. In contrast, individuals that later became subordinate appeared ‘bolder’ during the first period, both regarding swimming distance and time spent in the centre in the novel object test. As manual observation could not identify any erratic movement, our hypothesis is that the shyer animals are displaying a nervous behaviour, looking for an escape route out from the novel arena, which has also been seen in zebrafish stressed from restraint [34]. After a while, the animals calm down but again try to escape when a roof or a novel object is introduced. In contrast, bolder animals may investigate the situation visually while remaining immobile and start to explore more closely only after the situation has been interpreted as safe, thus showing higher activity in periods 2 and 3. Similar results were found by Schjolden *et al* [18] in rainbow trout selected for high (HR) or low (LR) stress response. In their study, the LR animals (less aggressive) moved more during the first two minutes after release into an open field, compared to the HR fish. However, the LR animals decreased their activity and during the last period the more aggressive HR line moved more [18].

In agreement with previous studies [28], once the social status had been established, it was not changed during the remainder of the experiment. Subordinates were always allowed to feed and the dominants did not defend the feed, suggesting stable but weak hierarchies. In contrast, social subordination in juvenile rainbow trout and Arctic charr (*Salvelinus alpinus*) often results in stress-induced anorexia along with chronically elevated plasma cortisol concentrations [35], [36], [37]. This strong difference between the species may be explained by the shoaling nature of the zebrafish while salmonids often are territorial during their parr stage.

Agonistic behaviour and fighting ability are known to be affected by several factors, e.g. size, sex, prior residence, energetic status and previous experience (the winner/loser effect) [38]. A repeatedly defeated animal may very well lose against an intruder half its size, if the intruder has no previous experience [39]. Similarly, animals that experience winning will fight much rougher and longer before the hierarchy is settled [39]. As the effect of previous experience, or winner-loser effect, is so strong, it was of particular interest to find that the dominant and subordinate individuals' behaviour was fully distinguished even before the social encounter. In order to minimise other effects that have been shown to affect the outcome, fish were reared in isolation for two weeks prior to being paired. Fish in a pair were also of the same sex and were introduced into an environment that was novel to them both. In addition, fish in pairs were of approximately equal size. As the animals were wild-caught, it has not been possible to adjust for differences in age and rearing environment.

In our experiment, each test lasted for 15 minutes but the first 5 min could distinguish between the calculated social statuses with only two individuals assigned wrongly. By adding the second period to the calculation, more than 89% of the variation in calculated status was explained by the model and none of the individuals were assigned to the wrong group. This shows that the model is valid even if a shorter time span is used. So in the future, only ten minutes of behavioural monitoring is required per test.

In agreement with Moretz *et al* [24], we found sex differences in bold behaviour that showed males to be bolder than females. Several previous studies have been unable to find any sex differences in zebrafish [24], [40], [41]. It was therefore interesting that higher values for most variables during the first period were associated with males, while higher values in the third period were typical for the females. This could be due to a decrease in activity of the males after the first period, an increase in the females in the last period, or possibly both. Due to large variation in behaviour, we have been unable to clarify which is the most likely, though this will be interesting for future studies.

We used three behavioural tests in which it was assumed that movement would be a good predictor of boldness, and that bold individuals would be more mobile [24]. In addition, it was assumed that bold individuals would spend more time in the centre, explore a novel object more and hide less under a shelter. The positive correlation between the time spent in the centre zones in the open field and novel object tests, as well as the negative correlation between distance moved in the open field test and time spent underneath the roof, support our expectations. However, there were also ambiguities in our results. Both distance moved in the novel object test and the roof test correlated negatively with distance moved in the open field test. One explanation could be that bolder fish inspect the roof and novel object from a distance, thus being bold in the open field test but more careful in the other tests. This is supported by the positive correlation seen between the distance moved in the roof test and the amount of time spent under the roof, which indicate that an explorative animal that moves around also passes under the roof more often. Taken together, this suggests that activity may not be a good measurement for boldness in all situations. Similar contradictions to the expected were also seen by Champagne *et al*, who showed that zebrafish stressed from confinement had a higher activity than controls in an open field, both in an inner and outer zone [34]. In our study, the time spent in the centre zone of the open field proved to be important, both in the distinction between dominant and subordinate individuals, as well as between the sexes. We therefore suggest that this may be a better predictor of bold behaviour than general activity.

In this study, we show that fish exhibit different behavioural profiles and that the outcome of a dyadic fight can be predicted from tests for boldness, with bolder individuals being more likely to become dominant.

## Supporting Information

Table S1 **Performed behavioural acts.** Mean ±SEM of raw data as well as of normalised data in parenthesis.
(DOC)
