# Correction: Boldness Predicts Social Status in Zebrafish (*Danio rerio*)

**DOI:** 10.1371/annotation/2fb5edb1-0100-45ef-b8bb-681cc1490295

**Published:** 2014-01-03

**Authors:** S. Josefin Dahlbom, David Lagman, Katrin Lundstedt-Enkel, L. Fredrik Sundström, Svante Winberg

There are two incorrect citations in section "4.1. Statistical Analysis." The first sentence of the second paragraph should have cited reference 32 instead of 33.

Additionally, in the second to last sentence of the second paragraph, reference 34 should have cited the following work:

Lundstedt-Enkel, K., Asplund, L., Nylund, K., Bignert, A., Tysklind, M., Olsson, M. and Örberg, J. (2006). Multivariate data analysis of organochlorines and brominated flame retardants in Baltic Sea guillemot (Uria aalge) egg and muscle. Chemosphere 65, 1591-1599. 

