# Supplementary material for: Boldness Predicts Social Status in Zebrafish (Danio rerio)
Source: PLoS One. 2011 Aug 17;6(8):e23565. doi: 10.1371/journal.pone.0023565 (PMC3157393; doi:10.1371/journal.pone.0023565)
Supplement: Table S1 — Performed behavioural acts. Mean ±SEM of raw data as well as of normalised data in parenthesis. (DOC) [file pone.0023565.s001.doc]

|  | Dominant | Subordinate | Female | Male |
| --- | --- | --- | --- | --- |
| Distance moved in open field- test | 3344±196 (52±7) | 2872±481 (52±10) | 3064±277 (46±5) | 3043±364 (53±6) |
| Distance moved in roof- test | 1737±453 (21±5) | 3943±2725 (32±10) | 2912±1194 (28±5) | 1759±416 (24±4) |
| Distance moved in novel object- test | 2044±435 (26±5) | 1035±432 (16±4) | 1741±302 (25±4) | 1527±344 (23±4) |
| Time spent in centre zone in open field- test | 28±3 (1±0.1) | 17±7 (0.6±0.3) | 15±3 (0.5±0.1) | 26±6 (1±0.2) |
| Time spent underneath the roof in roof- test | 248±102 (9±4) | 181±107 (7±4) | 210±66 (8±2) | 68±27 (3±1) |
| Time spent in centre zone in novel object- test | 34±30 (1±1) | 3±3 (0.1±0.1) | 29±18 (1±0.7) | 7±4 (0.2±0.2) |
